# Fostering Shared Decision-Making Between Patients and Health Care Professionals in Clinical Practice Guidelines: Protocol for a Project to Develop and Test a Tool for Guideline Developers

**DOI:** 10.2196/57611

**Published:** 2024-11-04

**Authors:** Lena Fischer, Fülöp Scheibler, Corinna Schaefer, Torsten Karge, Thomas Langer, Leon Vincent Schewe, Ivan D Florez, Andrew Hutchinson, Sheyu Li, Marta Maes-Carballo, Zachary Munn, Lilisbeth Perestelo-Perez, Livia Puljak, Anne Stiggelbout, Dawid Pieper

**Affiliations:** 1 Institute for Health Services and Health System Research, Faculty of Health Sciences Brandenburg Brandenburg Medical School (Theodor Fontane) Rüdersdorf Germany; 2 Center for Health Services Research Brandenburg Medical School (Theodor Fontane) Rüdersdorf Germany; 3 National Competency Center for Shared Decision Making University Hospital Schleswig-Holstein Kiel Germany; 4 SHARE TO CARE, Patient-Centered Care GmbH Cologne Germany; 5 German Agency for Quality in Medicine Berlin Germany; 6 CGS Clinical Guideline Services GmbH Berlin Germany; 7 German Network for Evidence-Based Medicine Berlin Germany; 8 Department of Pediatrics University of Antioquia Medellin Colombia; 9 Pediatric Intensive Care Unit Clínica Las Américas-AUNA Medellin Colombia; 10 School of Rehabilitation Science McMaster University Hamilton, ON Canada; 11 National Institute for Health and Care Excellence (NICE) Manchester United Kingdom; 12 Department of Endocrinology and Metabolism, Cochrane China Centre, MAGIC China Centre, Chinese Evidence-Based Medicine Centre West China Hospital, Sichuan University Chengdu China; 13 General Surgery Department Hospital Público de Verín Ourense Spain; 14 Department of General Surgery University of Santiago de Compostela Santiago de Compostela Spain; 15 Deontological Committee of the College of Physicians of Ourense Ourense Spain; 16 Healthcare Ethics Committee of Ourense Ourense Spain; 17 Health Evidence Synthesis, Recommendations and Impact (HESRI), School of Public Health University of Adelaide Adelaide Australia; 18 Evaluation Unit (SESCS) Canary Islands Health Service (SCS) Tenerife Spain; 19 Network for Research on Chronicity, Primary Care, and Health Promotion (RICAPPS) Tenerife Spain; 20 Spanish Network of Agencies for Assessing National Health System Technologies and Performance (RedETS) Tenerife Spain; 21 Center for Evidence-Based Medicine and Healthcare Catholic University of Croatia Zagreb Croatia; 22 Medical Decision Making, Department of Biomedical Data Sciences Leiden University Medical Center Leiden Netherlands; 23 Erasmus School of Health Policy and Management Erasmus University Rotterdam Rotterdam Netherlands

**Keywords:** shared decision-making, practice guidelines as topic, decision support techniques, support, decision-making, decisions, tool, testing tool, protocol, medical decision-making, patient decision aid, decision aid, tool development

## Abstract

**Background:**

Clinical practice guidelines (CPGs) are designed to assist health care professionals in medical decision-making, but they often lack effective integration of shared decision-making (SDM) principles to reflect patient values and preferences, particularly in the context of preference-sensitive CPG recommendations. To address this shortcoming and foster SDM through CPGs, the integration of patient decision aids (PDAs) into CPGs has been proposed as an important strategy. However, methods for systematically identifying and prioritizing CPG recommendations relevant to SDM and related decision support tools are currently lacking.

**Objective:**

The aim of the project is to develop (1) a tool for systematically identifying and prioritizing CPG recommendations for which SDM is considered particularly relevant and (2) a platform for PDAs to support practical SDM implementation.

**Methods:**

The project consists of 6 work packages (WPs). It is embedded in the German health care context but has an international focus. In WP 1, we will conduct a scoping review in bibliographic databases and gray literature sources to identify methods used to foster SDM via PDAs in the context of CPGs. In WP 2, we will conduct semistructured interviews with CPG experts to better understand the concepts of preference sensitivity and identify strategies for fostering SDM through CPGs. WP 3, a modified Delphi study including surveys and focus groups with SDM experts, aims to define and operationalize preference sensitivity. Based on the results of the Delphi study, we will develop a methodology for prioritizing key questions in CPGs. In WP 4, the tool will be developed. A list of relevant items to identify CPG recommendations for which SDM is most relevant will be created, tested, and iteratively refined, accompanied by the development of a user manual. In WP 5, a platform for creating and digitizing German-language PDAs will be developed to support the practical application of SDM during clinical encounters. WP 6 will conclude the project by testing the tool with newly developed and revised CPGs.

**Results:**

The Brandenburg Medical School Ethics Committee approved the project (165122023-ANF). An international multidisciplinary advisory board is involved to guide the tool development on CPGs and SDM. Patient partners are involved throughout the project, considering the essential role of the patient perspective in SDM. As of February 20, 2024, we are currently assessing literature references to determine eligibility for inclusion in the scoping review (WP 1). We expect the project to be completed by December 31, 2026.

**Conclusions:**

The tool will enable CPG developers to systematically incorporate aspects of SDM into CPG development, thereby providing guideline-based support for the patient-practitioner interaction. Together, the tool for CPGs and the platform for PDAs will create a systematic link between CPGs, SDM, and PDAs, which may facilitate SDM in clinical practice.

**International Registered Report Identifier (IRRID):**

DERR1-10.2196/57611

## Introduction

### Background

Clinical practice guidelines (CPGs) are systematically developed statements that provide evidence-based recommendations for decision-making by health care professionals and patients regarding appropriate care for particular health problems [1,2]. However, CPG recommendations should be contextualized to a patient’s personal circumstances, making it essential to apply a person-centered CPG approach [3,4]. Therefore, CPGs aim to support medical decision-making that considers the values and preferences of patients [4].

Shared decision-making (SDM) is an established procedure that supports patients and health care professionals in making joint decisions on health issues [5,6]. To reach a shared decision, discussing the possible options and supporting the patient to become aware of their individual preferences are essential SDM steps to be considered in practice [6,7].

SDM is desired by patients and expected from health care professionals when implementing CPG recommendations [8]. However, initial studies indicate that in their current form, CPGs are only suitable to a very limited extent for supporting health care professionals in engaging in SDM [9-11]. Additionally, data from implementation research and health care professionals’ practical experience with CPGs indicate that guideline-associated barriers impede the implementation of SDM in clinical practice: First, there commonly exists a risk that health care professionals misunderstand CPG recommendations as strict instructions to follow in any case, regardless of patient preferences [4,12]. A survey among health care professionals revealed that they perceive stronger and weaker recommendations as equally binding [13]. Second, depending on the health care system mode, the development of guideline-based quality indicators and the resulting nonfinancial and financial incentives for a health care facility (eg, certification and remuneration) can contribute to health care professionals following a recommendation rather than discussing the available treatment options with patients [4,14]. In particular, this applies to possible legal consequences such as malpractice claims [15]. From the perspective of SDM researchers, CPGs have been repeatedly criticized for hindering rather than promoting person-centered decision-making [4,11,16,17].

To overcome these barriers, it is necessary to integrate tools and use a variety of strategies, which enable SDM through CPGs and facilitate the implementation of SDM in clinical practice [4,5]. Therefore, it is important to first identify the CPG recommendations that are particularly relevant to SDM [4,7,18]. Evidence suggests that SDM is appropriate when faced with preference-sensitive options, where the choice of option depends largely on the individual preferences of the patient [4,5,7]. Preference-sensitive decisions are typically decisions in which there exist multiple options, but either the value that patients place on the associated benefits and harms differ or the available options are relatively balanced in terms of their benefits and harms [4,7]. Regarding the Grading of Recommendations, Assessment, Development and Evaluation methodology, conditional recommendations imply discussion of benefits and harms with patients [19]. However, it must be emphasized that the degree of preference sensitivity does not necessarily depend on the certainty of evidence.

When faced with such preference-sensitive decisions, decision support tools can be used to facilitate decision-making [4,18]. Decision support tools include, for instance, patient versions of CPGs, decision trees, and patient decision aids (PDAs) [4]. However, of these tools, particularly PDAs used during clinical encounters have the purpose of enabling SDM [4]. PDAs are available in various formats, such as booklets, leaflets, videos, or web-based resources [20]. In this project, we focus on PDAs that meet least the following elements [21-25]: (1) they provide evidence-based information about available treatment options, including their potential benefits and harms, to a specific patient target group and (2) they aim to facilitate decision-making and encourage SDM by empowering patients to explore their values and preferences regarding the available options.

PDAs cover different types of decisions (eg, prevention, diagnosis, and treatment) and are available for many different conditions and health problems (both acute and chronic) [22,25].

By establishing a more robust link between CPGs and PDAs, it is possible to translate general CPG recommendations into personalized recommendations for SDM processes more effectively [4,16]. Overall, there is consensus that PDAs can be a vehicle by which CPGs and SDM can be linked [4,26].

In addition, there is high-quality evidence that PDAs as a tool for SDM can improve patient-relevant outcomes [20,27]. However, research indicates that despite the proven benefits of high-quality PDAs, they are not being used enough in practice likely due to inadequate resources for continuous updating or because of health care professionals’ reluctance to accept them when not having been involved in their development [28-30]. In contrast, studies show that health care professionals consider brief, guideline-based PDAs to be valuable for practice [12,31].

At expert workshops hosted by the German Network for Evidence-Based Medicine (EbM-Network; on September 1, 2022, as part of the EbM Congress) and the Guidelines International Network (GIN; on September 24, 2022, as part of the GIN Conference) [32], national and international CPG developers unanimously formulated a high need for a tool that could aid in the structured prioritization of CPG recommendations according to their relevance for SDM as well as for a repository or database for guideline-based PDAs. Currently, there are no tools available for the systematic identification and prioritization of CPG recommendations with high SDM relevance [4].

### Objectives

We aim to develop a tool that will enable CPG developers to systematically identify and prioritize CPG recommendations for which SDM is considered particularly relevant. The tool for CPGs will be generic and can be used regardless of the underlying condition or health problem. In parallel, we aim to develop a platform for PDAs. While we focus on German CPGs for practical reasons, the project has a clear international focus and aims to inform CPG development worldwide.

## Methods

### Overview

The project, titled Development and testing of a tool to foster shared decision-making in clinical practice guidelines [Entwicklung und Testung eines Instruments zum Einbezug von Shared Decision-Making in Leitlinien; acronym EDELL], is funded by the German Innovation Fund and affiliated with the Brandenburg Medical School Theodor Fontane in cooperation with 3 consortium partners, including the German Agency for Quality in Medicine, Clinical Guideline Services GmbH, and SHARE TO CARE Patient-Centered Care GmbH. The guidelines working group of the German EbM-Network supports the project as a cooperation partner.

### Study Design

**Figure 1:**
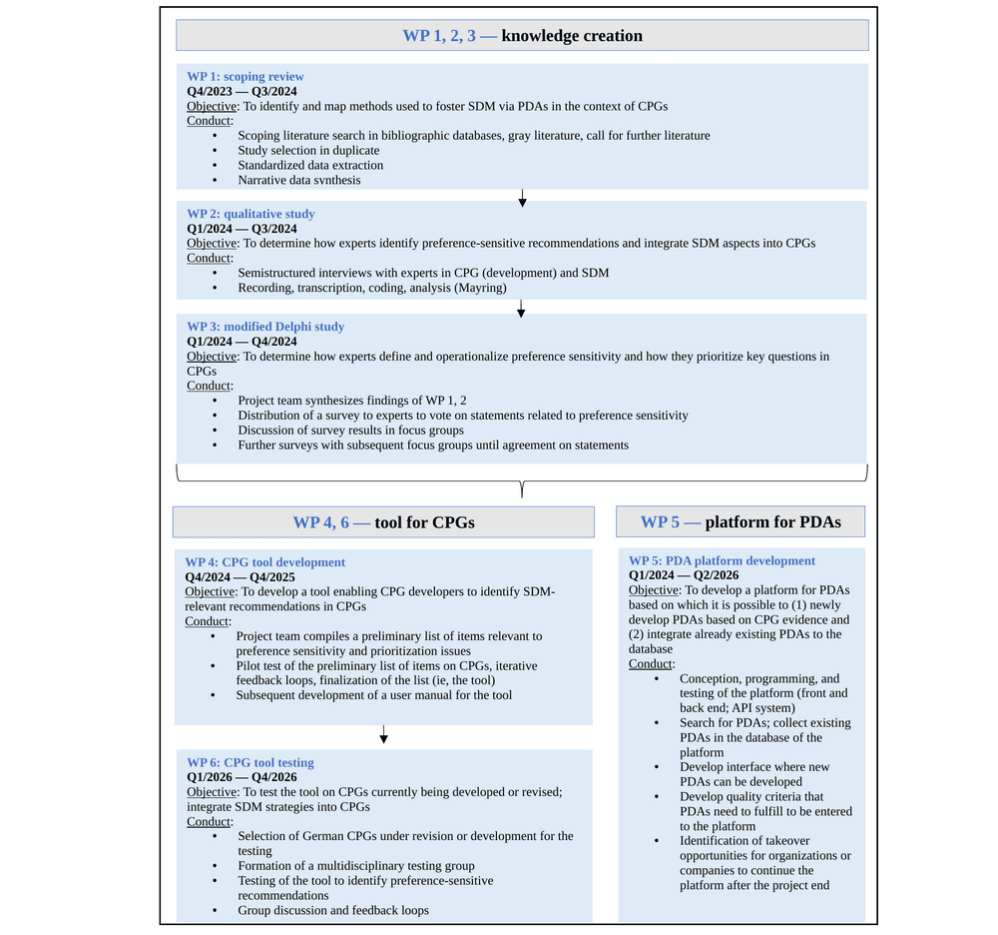
Overview of the project course. API: application programming interface; CPG: clinical practice guideline; PDA: patient decision aid; Q: quarter; SDM: shared decision-making.

The project comprises 6 work packages (WPs) and will run over 3 years (Q4 2023 to Q4 2026; Figure 1). WPs 1 to 3 serve as preparation for developing and testing the tool afterward in WPs 4 and 6. WP 5 runs parallel to the other WPs by developing a platform for PDAs to complement the tool.

In WP 1, we aim to summarize the methods currently used to foster SDM via PDAs in the context of CPGs. In WP 2, we will conduct interviews with international experts to determine how preference-sensitive recommendations can be identified and how SDM can be successfully implemented in CPGs. In WP 3, a modified Delphi study with SDM experts will be conducted, aiming to define and operationalize preference sensitivity and develop a methodology for prioritizing key questions in CPGs. WP 4 will concern the actual development of the tool by generating items that are relevant for the identification of SDM-relevant recommendations. Subsequent evaluation of the tool’s first version will follow. In WP 5, our aim is to develop a digital platform that will provide (1) a toolkit for the development of PDAs and (2) a database for German language PDAs. Finally, in WP 6, practical testing of the tool within selected CPGs will follow.

### WP 1: To Identify and Map Methods Used to Foster SDM Through PDAs in the Context of CPGs—A Scoping Review

#### Objective, Literature Search, and Eligibility Criteria

In WP 1, we will conduct a scoping review, which adheres to the PRISMA-ScR (Preferred Reporting Items for Systematic Reviews and Meta-Analysis Extension for Scoping Reviews) [33] and follows the updated methodological guidance for the conduct of scoping reviews proposed by the JBI (formerly Joanna Briggs Institute) [34].

To provide an overview of methods to foster SDM via PDAs in the CPG development process, we will use the GIN Public Toolkit—Patient and Public Involvement in Guidelines as a starting point [4]. To account for the progress after the publication of the GIN Public Toolkit, we will update the current state of knowledge by conducting a scoping review in relevant scientific databases (PubMed and Embase; see Multimedia Appendix 1 for full search strategy). To account for research-relevant information that is not formally published [35], we will additionally search in Google and Google Scholar. The search will be restricted to studies published since 2000, as the link between CPGs and PDAs is relatively new [3,4]. References will be eligible for inclusion if they meet all the following criteria: they are published from the year 2000 onward, provide information about the methodology of developing and integrating PDAs in the context of CPGs, are available in German or English, and have full texts available. The exclusion will apply to studies not focusing on PDAs but on other patient-directed knowledge tools, such as the patient version of CPGs.

As part of the scoping review, handbooks, method manuals, and other documents of international CPG organizations that are known for providing patient-directed knowledge tools [36] (see Multimedia Appendix 2 for a list of organizations) will be sought via their websites. To obtain additional information on methods for the development and integration of PDAs in the context of CPGs, we will contact experts via the earlier-mentioned CPG organizations [36] and via GIN. Ideally, this will help to identify CPGs that have already systematically integrated PDAs, as the applied methods for doing so cannot be easily identified using other research techniques. After removing potential duplicates, 2 reviewers (LF and LVS) will independently screen all identified literature based on title and abstract for eligibility. They will independently continue with a full-text screening of references found to be eligible at the title and abstract screening stage. A list with the reasons for excluding full texts will be compiled, while disagreements will be resolved through discussion and by contacting a third reviewer (DP) for advice.

#### Data Collection and Analysis

One reviewer (LF) will extract findings into standardized tables, which the second reviewer (LVS) will double-check. The extraction tables will contain categories of general study and method manual characteristics, such as title, publication year, authors, and their corresponding CPG organization. Additionally, information concerning the methods for developing and integrating PDAs in the context of CPGs will be extracted. Disagreements will be again resolved through discussion, and consensus will be reached by the judgment of a third reviewer (DP). The generated information will be narratively synthesized.

### WP 2: To Determine How Experts Identify Preference-Sensitive CPG Recommendations and Integrate SDM Strategies Into CPGs—A Qualitative Study

#### Objective

In WP 2, we aim to explore experts’ perspectives on the identification of preference-sensitive CPG recommendations and the successful integration of SDM strategies into CPGs by conducting a qualitative study via semistructured interviews. We will use COREQ (Consolidated Criteria for Reporting Qualitative Research) for planning and reporting [37].

#### Sampling

For recruitment, we will develop a sample plan to ensure that relevant experts of national and international CPG organizations are adequately presented (ie, selective sampling). By contacting the 17 CPG organizations mentioned earlier [36] and potential other identified relevant organizations, we will continue to recruit experts until we reach data saturation. However, we expect that 10-15 experts will be sufficient to reach data saturation. Their expertise concerns the areas of CPGs, PDAs, and SDM. For example, experts can be either methodologists or health care professionals involved in CPG development.

#### Interview Guide

The consortium and all collaborators will conceptualize an interview guide before the interview. The developed interview guide will be piloted in the first interviews and adapted if necessary. The guide will include preformulated open questions related to the project topic but will focus in particular on questions concerning (1) What is meant by preference sensitivity? and (2) How can SDM be successfully integrated into CPGs? All interviewees will receive the interview guide in advance.

#### Data Collection and Analysis

The interviews will be conducted by telephone or videoconference and digitally recorded. The interviewer will take notes during or immediately after the interview. Conducted interviews will be transcribed, coded, and analyzed using MAXQDA analysis software (standard version 18.2.4; Verbi GmbH). The interviews will be analyzed using qualitative content analysis according to Mayring [38]. A category system will be developed deductively based on the core themes or questions. Further subcategories may be developed inductively. Category building will be discussed within the research team.

### WP 3: To Determine How Experts Define and Operationalize Preference Sensitivity and How They Prioritize Key Questions in CPGs—Modified Delphi Study

#### Objective

WP 3 will start with a workshop to synthesize the results of WP 1 and preliminary results of WP 2, based on which a multistage Delphi study including Delphi focus groups will be conducted. As there has been limited research in the area of preference sensitivity and prioritization of CPG questions with regard to the provision of PDAs, WP 3 aims (1) to provide a clear and generic definition of preference sensitivity and (2) to identify clear criteria for prioritizing CPG recommendations where PDAs may be required. To systematically develop statements on the topics of preference sensitivity and prioritization of CPG questions regarding the provision of PDAs, all members of the core team and the patient partners of the project will participate in the workshop. The subsequent modified Delphi study will be based on the internationally proven RAND/UCLA Appropriateness Method [39]. We will use ACCORD (Accurate Consensus Reporting Document) for reporting [40].

#### Sampling

Expert panelists will be recruited through GIN and the International Shared Decision Making Society and invited by email to participate in the Delphi study. We will select the panelists on the basis of their expertise while ensuring representation of relevant perspectives (ie, selective sampling of CPG or SDM methodologists and health care professionals). The Delphi survey rounds will each consist of 20-30 participants, approximately half of whom will actively participate in the Delphi focus groups.

#### Delphi Process

Based on the workshop conducted in advance, statements on the topic of preference sensitivity will be developed and put to vote. Before distributing the survey to the panelists, the developed statements will be pilot-tested by the Brandenburg Medical School Theodor Fontane and consortium partners to ensure clarity, usability, and functionality. As a first step in the Delphi study, a group of national SDM experts will evaluate and, if necessary, expand criteria that are considered appropriate for SDM. In the second step, international panelists will be asked to consider and vote on the inclusion of statements in web-based Delphi rounds in order to achieve a high level of agreement. Voting will take place on a scale from 1=no agreement to 8=full agreement. To specify the statements for the second Delphi round and the Delphi focus group discussion, a free-text option will be offered for each item. For a statement to be accepted, 75% (n=15-23) of the participants must agree with the statement, defined as a value of 5 or higher. Participants in the Delphi rounds will be sent the respective preliminary results and asked for feedback. As the number of Delphi rounds is difficult to estimate a priori, we will flexibly adapt the number of rounds needed to reach an agreement for statements put to vote. However, most Delphi studies reach agreement within 2 or 3 rounds, which is also assumed to be the case here. A new vote will be taken in each round.

In addition to the written feedback, the Delphi focus group will be held via videoconference. The purpose of the focus group is to reach an agreement on statements that were not agreed upon in the previous survey rounds. With an agenda, background information, consent form, and survey results provided in advance, participants will discuss the statements before a verbal summary and final vote. The results of this Delphi focus group will be recorded and incorporated into the methods to be developed.

### WP 4: To Develop a Valid and Reliable Tool Enabling CPG Developers to Identify SDM-Relevant Recommendations in CPGs—Item Generation, User Manual, and Testing

#### Objective

The findings from the previous WPs will form the basis for the development of the tool to identify recommendations in CPGs that are most relevant to SDM. The methodology for developing the tool is based on the methods from the AGREE II (Appraisal of Guidelines for Research and Evaluation II) portfolio [41]. The process that we will follow, as described below, is geared on the development of the AGREE-REX (Appraisal of Guidelines for Research and Evaluation-Recommendations Excellence) [42,43]. A small working group (consisting of the project partners) will compile a preliminary list of initially relevant items, which will mainly be derived from the results of WPs 1-3. Subsequently, the list will be revised and reduced in terms of focus and objectives as well as potential overlaps between items. At the same time, a user manual will be produced, presenting the items with background, rationale, and explanation.

#### Items

Items will reflect relevant aspects of preference sensitivity as identified in WPs 1, 2, and 3 as well as aspects of prioritization such as relevance of the clinical decision, burden of disease or treatment, and others. Items will be assessed using a Likert scale. An overall score may be calculated by summing up the individual scores. The use of weighing different items will be assessed by the development team. The higher the total score, the greater the need to provide SDM support for the respective recommendation. The possibility to generate a total score will facilitate the use and interpretation of the tool for subsequent application.

#### Prioritization

The number of PDAs developed or integrated into a CPG will depend highly on the resources available. The tool will reflect this by providing the possibility to prioritize those recommendations for which decision support seems most needed. Whether a cut-off value or a ranking according to total scores is more helpful will have to be considered.

#### Testing

The items on the list (ie, the preliminary tool), including the user manual, will be tested on 5 completed CPGs within the working group and then revised. We will ensure that the selected CPGs cover a broad spectrum of (1) conditions and (2) areas of application (diagnosis, prevention, and treatment).

The tool will be applicable to all recommendations of a CPG, regardless of the grade of recommendation. However, it may be discussed whether strong negative recommendations, probably recommendations for emergency settings, are worth rating, given that the addressed intervention is no sensible alternative (no benefit but relevant harm) or the urgency to act does not allow for SDM. The resulting version of the tool will be circulated among the project partners. They will be asked to provide feedback with regard to clarity, completeness, relevance, and user-friendliness. This includes both the items and the overall structure. The group will use the feedback received to revise the tool. This version will then be circulated to the project partners to test the tool against 2 CPGs and provide feedback, which will be followed by another revision and refinement loop. The project partners will finalize the items in a concluding workshop.

### WP 5: To Develop a Platform for PDAs

#### Objective

A key strategy to promote SDM in CPGs is to attach or link PDAs to SDM-relevant recommendations so that health care professionals can directly find the PDA and use it as support for the SDM process when their patient is facing a medical decision. In addition, there is currently no platform in Germany that (1) collects already existing PDAs, (2) makes them easy to find, (3) systematically compiles them, and (4) facilitates the creation of machine-readable PDAs through a user-friendly web-based editor. Taken together, we recognized the need to develop a platform for PDAs. With this platform, it will be possible to develop PDAs semiautomatically and to digitize as many of the existing formats of German-language PDAs as possible in a database in order to make them ubiquitously available via an interface for other applications (eg, hospital or physician information systems). The aim is to create a valuable possibility, in particular for those who create CPGs, to link existing PDAs and create new PDAs on key issues with little effort from the evidence syntheses that have been done in advance as part of the CPG work. WP 5 is of central importance for the project, as the development of the tool for CPGs alone misses the point of care practice. Without such a platform, the CPG tool would be able to identify and prioritize the recommendations for which SDM seems most relevant in the CPG development process, but the bridge to support the practical SDM process between health care professionals and patients would be missing. The platform also corresponds with the idea that all CPGs should be digitized so that PDAs can ultimately be better integrated or linked with CPGs and thus be more readily available at the point of care. An example of such a platform is PADA (formerly Collaborative Platform for Authors of Healthcare Decision Aids), which can be used to create new PDAs [44]. However, our target database aims to take another step forward by integrating already available PDAs, which may also differ in their formats, into the database.

#### Platform Development

To this end, a data structure is being developed that can be used to map the majority of existing PDA formats and make them digitally available. As a central function, the database will allow PDAs to be linked to each other and to external resources, for example, to the respective recommendation in the CPG and vice versa, when possible.

Three forms of presentation for PDAs are planned for the first version of the platform: (1) digital user-friendly option overviews, (2) multistage and partially digital user-friendly PDAs, and (3) option for information based exclusively on a sequence of texts, tables, and graphics.

To fill the database, known authors of PDAs will be contacted and invited to enter their PDAs in the German language. Additionally, a simultaneous search will be conducted in bibliographic databases such as PubMed and Embase, complemented by a Google search. A selection of PDAs identified in this way will be entered into the database when agreed by the PDA authors. The PDAs will be selected according to quality criteria defined during the project phase.

For the specific implementation of important functions, such as import, maintenance, linking of literature sources, data structures for study results or effect measures, and word export, previous knowledge of the consortium partners can be used. The platform should allow registered authors to independently enter PDAs into the system. For this purpose, a back end will be developed that includes a team-based user administration, an input system for PDAs in the 3 modes described earlier, as well as a release workflow for the PDAs and a versioning system for tracking changes. A literature management system for citing literature references in the PDA and a glossary function are also planned.

The front end with the interface for searching and retrieving PDAs should include a simple and comprehensive search with filter options using keywords and metadata (eg, subject area, year, and publisher). Users of the platform should be able to rate and review the PDAs (nonpublic). Possible improvements in presentation and completeness can be suggested by automated feedback mechanisms aimed at continuously evaluating and improving the quality of PDAs. The interface will be developed in a responsive design, making it possible to be displayed on mobile devices, tablets, and PCs. In addition, it should be possible to export the PDA as a PDF and other formats.

The system should provide an interface (application programming interface), through which third-party systems can access the system and retrieve PDAs. For example, integration into hospital information systems, decision support systems, or similar would be possible. The interface should have an authentication mechanism and allow access to the PDA and its metadata in a structured form.

To meet the needs of CPG developers and patients, they will be involved in the planning and subsequent test phase. To ensure the continuation of the platform after the end of the funding phase, it is intended to evaluate possibilities for further development and continued operation. A viable concept for this will be developed during the funding phase. In our view, possible institutions that could be interested in the continuation of such a platform include the EbM-Network, the German Network for Health Literacy, and the Association of the Scientific Medical Societies (AWMF), in addition to their digitization strategy.

#### Platform Quality Criteria

For the integration of PDAs into the platform, inclusion criteria, in terms of minimum requirements, will be developed in advance and applied to the identified PDAs. The Good Practice Health Information of the EBM-Network [45] and the International Patient Decision Aid Standards criteria [46], among others, will be used to develop the quality criteria.

### WP 6: To Test the Tool on CPGs Currently Being Developed or Revised

#### Objective

The last WP will serve to test the tool on approximately 12-15 German CPGs. Applying the tool in practice will allow to gain insights into its handling and application. At the same time, the actual integration of PDAs to CPG recommendations identified by the tool as sensitive to individual preferences will be realized.

#### Selection of Suitable CPGs

For pragmatic reasons, the selected CPGs should be close to completion in order to test the developed tool. It is a prerequisite that the CPG group is currently working on the CPG, but at the same time, new or updated CPG recommendations have already been established so that individual recommendations or entire CPG chapters can already be used. Given the increasing number of living guidelines [47], this selection process seems realistic and has the advantage of not having to wait for the entire CPG to be finalized or updated. We will consider S3 CPGs (which represent the highest level of a systematic approach in the CPG development process according to the AWMF) registered in the AWMF registry or CPGs under revision, including the National Program for Disease Management Guidelines (NDMG) and CPGs of the German Guideline Program in Oncology (GGPO). For this purpose, an overview of these CPGs will be obtained from the AWMF registry and sorted by planned publication date (or alternatively expiry date of the CPG to be updated). In this way, the CPG organizations that are closest to finalizing the respective CPG will be contacted first. The relevant CPG organizations will be contacted consecutively but in such a way that at least 3 CPGs of the NDMG and GGPO will be included. Pragmatic aspects, such as the willingness to participate in the testing of the tool, will be particularly important in the selection process. The number of CPG recommendations included in the testing will vary from chapter to chapter, both within and across the CPGs. Approximately 30 recommendations per CPG should be included in the test. To achieve the planned number of recommendations for testing the tool, we may also decide to use several chapters of a CPG.

#### Testing Phase

Similar to the development of quality indicators [48] or patient versions of CPGs [49] during the initial CPG development process, a small multidisciplinary team will be formed for each CPG, on which the tool will be tested. For this purpose, in addition to the person responsible for the respective CPG chapter (ie, CPG methodologist and health care professional), the involvement of patients and patient partners is planned. Patients or patient partners who are already involved in the CPG development will be contacted first. Otherwise, alternatives will be sought through patient organizations. The team will be completed by a person involved in the development of the tool (ie, one member of our project team) and an SDM expert. At the beginning of the testing phase, the latter 2 group members will present the SDM concept and the developed tool to the multidisciplinary team. Using the tool, the multidisciplinary team approaches a CPG that is being revised or newly developed and screens all CPG recommendations. This means that the tool will be applied to every single recommendation. Ratings will be given by health care professionals and patients. These ratings will be used to prioritize preference-sensitive recommendations. The SDM expert and a member of our project team will support the rest of the team in gathering continuous feedback with the intention of refining the tool during the application phase, if necessary. Each test group will provide (1) verbal feedback directly during the testing phase and (2) written feedback via a questionnaire after the testing phase.

In a final workshop, the findings and experiences will be reflected upon with representatives of the NDMG, GGPO, and AWMF. The aim of the workshop (or several workshops, if necessary) is to implement the tool in the respective manuals of the CPG organizations, taking into account their digitization strategies.

### Ethical Considerations

For the qualitative studies (ie, WPs 2 and 3), ethics approval was given by the Brandenburg Medical School on January 29, 2024 (165122023-ANF). We will obtain informed consent from all participants prior to conducting these WPs, and participants will have the opportunity to opt out at any point in time without giving reasons. Data are deidentified and cannot be traced back to individuals. Participants in WPs 2 and 3 will not receive any compensation; patient partners in the project will receive an honorarium to compensate for the time they devote to the project. We will conduct our research in accordance with the Declaration of Helsinki, respect the General Data Protection Regulation and findability, accessibility, interoperability, and reusability principles for data management [50]. Ethics approval is not required for the other WPs, as we will not be collecting personal data. In WP 1, the scoping review involves secondary research based on already published material, whereas WPs 4, 5, and 6 focus on technical development and testing processes that will be directly overseen by our project team. The findings from each WP of the project will be published in peer-reviewed journals and presented at scientific conferences. Results will also be made available early in prepublication repositories.

## Results

The project has started on December 1, 2023, and is ongoing. To ensure that the patient perspective is taken into account, patient partners are involved throughout the project and provide input for each WP. For example, patient partners will be consulted when compiling the list of items as part of the tool development in WP 4 or when setting up and designing the platform for PDAs in WP 5. As the project has international relevance, an international advisory board is convened. The members of the advisory board are internationally recognized experts in the field of CPGs and SDM. As of February 20, 2024, we are currently assessing literature references to determine eligibility for inclusion in the scoping review (WP 1). Upon completion of WP 1 and WP 2, we expect to provide key methodological considerations that will enable the linked development of CPGs and guideline-based PDAs. At the end of WP 3, a methods paper on the definition and operationalization of preference sensitivity and the prioritization of CPG key questions for PDAs will form the basis for the subsequent WP 4. Once developed in WP 4 and tested in WP 6, the tool for CPGs will provide a methodologically valid identification of CPG recommendations for which SDM appears to be particularly relevant during the CPG development process. The platform for PDAs (WP 5) translates the objective of supporting SDM into practice by providing tools to support joint decision-making between patients and health care professionals. The PDAs on the platform can also be used to link to the recommendations in the CPG that the tool has identified as most relevant for SDM. We expect all parts of the project to be completed by December 31, 2026.

## Discussion

### Main Findings

This protocol outlines a multistage project aimed at developing a tool for CPGs to systematically identify and prioritize CPG recommendations for which SDM is particularly relevant. It describes the parallel creation of a platform for PDAs to support the application of SDM in clinical practice. It outlines how both the tool for CPGs and the platform for PDAs can work together to foster SDM between patients and health care professionals.

### The Project in Context

To our knowledge, this is the first project to provide a tool for CPG developers to systematically consider SDM during CPG development. The tool addresses the issue that current CPGs often do not adequately facilitate deviations from recommendations when appropriate [9]. By using the tool, CPG recommendations that are particularly sensitive to individual patient preferences can be highlighted, helping health care professionals apply CPGs correctly and practice SDM. The outputs of each WP will be able to support both evidence-based and person-centered care [51,52].

Drawing on the proven effectiveness of PDAs [22], we anticipate that the platform for PDAs has the potential to improve decision-making by providing a digital toolkit for the development of guideline-based PDAs while serving as a central repository for high-quality PDAs at the same time. This project is in line with international efforts to support a person-centered approach to CPGs [53] as well as efforts to use digital support for the development of CPGs and PDAs (eg, computable guidelines [54] and MAGICapp [55]). In the broader health care context, this project represents one of several strategies needed to strengthen SDM in practice [27].

### Implications During and After the Project Phase

Although the aim is to develop a generic tool, we recognize that during the development process, it may become apparent that the tool needs to be adapted or modified due to the wide range of conditions or health problems addressed in CPGs. We will test the tool during the ongoing update or creation of CPGs. This will have the advantage that the tool can be tested directly in practice but will be accompanied by the difficulty that CPG development groups will be confronted with an increased workload. While the tool will only be developed and tested on CPGs in the project phase, we anticipate that these efforts will also lead to the integration of PDAs into patient versions of CPGs.

Input from an international advisory board that collaborates across countries to advise on the development of the tool will ensure an international perspective on CPGs and SDM. Once translated, the tool for CPGs will also be available in multiple languages. We plan to keep updating, maintaining, and disseminating the tool after this project. Further quantitative validation will also be helpful. With regard to PDAs, more research appears to be needed on issues such as “Which type or format of PDA is most effective (for which patient group)?” “When to best use it?” and “What is its impact, depending on the type of decision, condition, and other patient-related factors (such as socioeconomic factors and health literacy)?”

### Limitations

Within the project, each WP has some limitations. These will be discussed further in the publication of each WP. In WP 1, limited resources may prevent the identification of all relevant documents, as we cannot search all international CPG organization websites. In WPs 2 and 3, a selective sample may not fully reflect all health care contexts and stakeholder perspectives, although we will mitigate this through careful recruitment at the international level. In WPs 4 and 6, we will develop and test the tool for CPGs primarily in the context of German CPGs. However, CPG recommendations may differ depending on the geographical context [56], and cultural influences may contribute to a different operationalization of preference sensitivity [57-59]. To overcome this shortcoming, the international project advisory board will be consulted for its expertise in all phases of the project. Similarly, the platform for PDAs (WP 5) will be limited to German-language PDAs for pragmatic reasons.

### Conclusions

This project will produce a generic tool to promote SDM as part of the CPG development process, with the potential to improve the quality of health care by enabling CPG developers to systematically incorporate aspects of SDM into CPGs. This will offer users, particularly health care professionals, guideline-based support for the patient-clinician interaction. The platform for PDAs will systematically link PDAs to SDM-relevant CPG recommendations, thereby facilitating the use of PDAs in practice. In the long term, this project intends to raise awareness among international CPG organizations to make SDM and related tools an integral part of CPGs and to foster international collaboration to improve SDM practices worldwide.

## Appendix

Multimedia Appendix 1 Search strategies for bibliographic databases.

Multimedia Appendix 2 List of clinical practice guideline organizations searched.

## Abbreviations

ACCORD: Accurate Consensus Reporting Document
AGREE II: Appraisal of Guidelines for Research and Evaluation II
AGREE-REX: Appraisal of Guidelines for Research and Evaluation-Recommendation Excellence
AWMF: Association of the Scientific Medical Societies
COREQ: Consolidated Criteria for Reporting Qualitative Research
CPG: clinical practice guideline
EbM-Network: German Network for Evidence-Based Medicine
GGPO: German Guideline Program in Oncology
GIN: Guidelines International Network
NDMG: National Program for Disease Management Guidelines
PDA: patient decision aid
PRISMA-ScR: Preferred Reporting Items for Systematic Reviews and Meta-Analysis Extension for Scoping Reviews
SDM: shared decision-making
WP: work package

## Data Availability

The datasets generated and analyzed during this study are available from the corresponding author on reasonable request.

## References

[1] Qaseem A, Forland F, Macbeth F, Ollenschläger G, Phillips S, van der Wees P, Board of Trustees of the Guidelines International Network (2012). Guidelines International Network: toward international standards for clinical practice guidelines. Ann Intern Med.

[2] (2020). AWMF-Regelwerk Leitlinien [AWMF manual guidelines]. Association of the Scientific Medical Societies (AWMF).

[3] Kühlein T, Schaefer C (2020). Leitlinien: Die Kunst des Abweichens [Guidelines: the art of deviation]. Dtsch Arztebl Int.

[4] Schaefer CA (2021). How to foster shared decision making through guidelines. Guidelines International Network.

[5] van der Weijden T, Boivin A, Burgers J, Schünemann HJ, Elwyn G (2012). Clinical practice guidelines and patient decision aids. An inevitable relationship. J Clin Epidemiol.

[6] Stiggelbout AM, Van der Weijden T, De Wit MPT, Frosch D, Légaré F, Montori VM, Trevena L, Elwyn G (2012). Shared decision making: really putting patients at the centre of healthcare. BMJ.

[7] van der Horst DEM, Garvelink MM, Bos WJW, Stiggelbout AM, Pieterse AH (2023). For which decisions is shared decision making considered appropriate?—A systematic review. Patient Educ Couns.

[8] Fearns N, Kelly J, Callaghan M, Graham K, Loudon K, Harbour R, Santesso N, McFarlane E, Thornton J, Treweek S (2016). What do patients and the public know about clinical practice guidelines and what do they want from them? A qualitative study. BMC Health Serv Res.

[9] Morgott M, Heinmüller S, Hueber S, Schedlbauer A, Kühlein T (2020). Do guidelines help us to deviate from their recommendations when appropriate for the individual patient? A systematic survey of clinical practice guidelines. J Eval Clin Pract.

[10] Maes-Carballo M, Muñoz-Núñez I, Martín-Díaz M, Mignini L, Bueno-Cavanillas A, Khan KS (2020). Shared decision making in breast cancer treatment guidelines: development of a quality assessment tool and a systematic review. Health Expect.

[11] Gärtner FR, Portielje JE, Langendam M, Hairwassers D, Agoritsas T, Gijsen B, Liefers G, Pieterse AH, Stiggelbout AM (2019). Role of patient preferences in clinical practice guidelines: a multiple methods study using guidelines from oncology as a case. BMJ Open.

[12] Carlsen B, Glenton C, Pope C (2007). Thou shalt versus thou shalt not: a meta-synthesis of GPs' attitudes to clinical practice guidelines. Br J Gen Pract.

[13] Nast A, Sporbeck B, Jacobs A, Erdmann R, Roll S, Sauerland U, Rosumeck S (2013). Study of perceptions of the extent to which guideline recommendations are binding: a survey of commonly used terminology. Dtsch Arztebl Int.

[14] Elwyn G, Price A, Franco JVA, Gulbrandsen P (2023). The limits of shared decision making. BMJ Evid Based Med.

[15] Mackey TK, Liang BA (2011). The role of practice guidelines in medical malpractice litigation. Virtual Mentor.

[16] Elwyn G, Wieringa S, Greenhalgh T (2016). Clinical encounters in the post-guidelines era. BMJ.

[17] Young CE, Boyle FM, Brooker KS, Mutch AJ (2015). Incorporating patient preferences in the management of multiple long-term conditions: is this a role for clinical practice guidelines?. J Comorb.

[18] van der Weijden T, Pieterse AH, Koelewijn-van Loon MS, Knaapen L, Légaré F, Boivin A, Burgers JS, Stiggelbout AM, Faber M, Elwyn G (2013). How can clinical practice guidelines be adapted to facilitate shared decision making? A qualitative key-informant study. BMJ Qual Saf.

[19] Guyatt GH, Oxman AD, Vist GE, Kunz R, Falck-Ytter Y, Alonso-Coello P, Schünemann HJ (2008). GRADE: an emerging consensus on rating quality of evidence and strength of recommendations. BMJ.

[20] Stacey D, Légaré F, Lewis K, Barry MJ, Bennett CL, Eden K, Holmes-Rovner M, Llewellyn-Thomas H, Lyddiatt A, Thomson R, Trevena L (2017). Decision aids for people facing health treatment or screening decisions. Cochrane Database Syst Rev.

[21] Elwyn G, O'Connor A, Stacey D, Volk R, Edwards A, Coulter A, Thomson R, Barratt A, Barry M, Bernstein S, Butow P, Clarke A, Entwistle V, Feldman-Stewart D, Holmes-Rovner M, Llewellyn-Thomas H, Moumjid N, Mulley A, Ruland C, Sepucha K, Sykes A, Whelan T (2006). Developing a quality criteria framework for patient decision aids: online international Delphi consensus process. BMJ.

[22] Stacey D, Lewis KB, Smith M, Carley M, Volk R, Douglas EE, Pacheco-Brousseau L, Finderup J, Gunderson J, Barry MJ, Bennett CL, Bravo P, Steffensen K, Gogovor A, Graham ID, Kelly SE, Légaré F, Sondergaard H, Thomson R, Trenaman L, Trevena L (2024). Decision aids for people facing health treatment or screening decisions. Cochrane Database Syst Rev.

[23] (2024). What are patient decision aids?. International Patient Decision Aid Standards (IPDAS) Collaboration.

[24] Elwyn G, Frosch D, Volandes AE, Edwards A, Montori VM (2010). Investing in deliberation: a definition and classification of decision support interventions for people facing difficult health decisions. Med Decis Making.

[25] Drug Therapeutics Bulletin (2013). An introduction to patient decision aids. BMJ.

[26] Bravo P, Härter M, McCaffery K, Giguère A, Hahlweg P, Elwyn G (2022). Editorial: 20 years after the start of international shared decision-making activities: is it time to celebrate? Probably…. Z Evid Fortbild Qual Gesundhwes.

[27] Scheibler F, Geiger F, Wehkamp K, Danner M, Debrouwere M, Stolz-Klingenberg C, Schuldt-Joswig A, Sommer CG, Kopeleva O, Bünzen C, Wagner-Ullrich C, Koch G, Coors M, Wehking F, Clayman M, Weymayr C, Sundmacher L, Rüffer JU (2024). Patient-reported effects of hospital-wide implementation of shared decision-making at a university medical centre in Germany: a pre-post trial. BMJ Evid Based Med.

[28] Stacey D, Suwalska V, Boland L, Lewis KB, Presseau J, Thomson R (2019). Are patient decision aids used in clinical practice after rigorous evaluation? A survey of trial authors. Med Decis Making.

[29] Elwyn G, Scholl I, Tietbohl C, Mann M, Edwards AGK, Clay C, Légaré F, van der Weijden T, Lewis CL, Wexler RM, Frosch DL (2013). "Many miles to go …": a systematic review of the implementation of patient decision support interventions into routine clinical practice. BMC Med Inform Decis Mak.

[30] Elwyn G, Légaré F, van der Weijden T, Edwards A, May C (2008). Arduous implementation: does the Normalisation Process Model explain why it's so difficult to embed decision support technologies for patients in routine clinical practice. Implement Sci.

[31] (2021). Evaluation der Nationalen VersorgungsLeitlinien: Abschlussbericht [Evaluation of the national health care guidelines: final report]. German Agency for Quality in Medicine (ÄZQ).

[32] Schaefer C (2022). How to develop guideline-based decision aids. GIN Conference.

[33] Tricco AC, Lillie E, Zarin W, O'Brien KK, Colquhoun H, Levac D, Moher D, Peters MD, Horsley T, Weeks L, Hempel S, Akl EA, Chang C, McGowan J, Stewart L, Hartling L, Aldcroft A, Wilson MG, Garritty C, Lewin S, Godfrey CM, Macdonald MT, Langlois EV, Soares-Weiser K, Moriarty J, Clifford T, Tunçalp Ö, Straus SE (2018). PRISMA Extension for Scoping Reviews (PRISMA-ScR): checklist and explanation. Ann Intern Med.

[34] Peters MDJ, Marnie C, Tricco AC, Pollock D, Munn Z, Alexander L, McInerney P, Godfrey CM, Khalil H (2020). Updated methodological guidance for the conduct of scoping reviews. JBI Evid Synth.

[35] Godin K, Stapleton J, Kirkpatrick SI, Hanning RM, Leatherdale ST (2015). Applying systematic review search methods to the grey literature: a case study examining guidelines for school-based breakfast programs in Canada. Syst Rev.

[36] Santesso N, Morgano GP, Jack SM, Haynes RB, Hill S, Treweek S, Schünemann HJ (2016). Dissemination of clinical practice guidelines: a content analysis of patient versions. Med Decis Making.

[37] Tong A, Sainsbury P, Craig J (2007). Consolidated Criteria for Reporting Qualitative Research (COREQ): a 32-item checklist for interviews and focus groups. Int J Qual Health Care.

[38] Mayring P (2021). Qualitative Content Analysis.

[39] Fitch K, Bernstein SJ, Aguilar MD, Burnand B, LaCalle JR, Lazaro P (2001). The RAND/UCLA Appropriateness Method User's Manual.

[40] Gattrell WT, Logullo P, van Zuuren EJ, Price A, Hughes EL, Blazey P, Winchester CC, Tovey D, Goldman K, Hungin AP, Harrison N (2024). ACCORD (ACcurate COnsensus reporting document): a reporting guideline for consensus methods in biomedicine developed via a modified Delphi. PLoS Med.

[41] Brouwers MC, Spithoff K, Lavis J, Kho ME, Makarski J, Florez ID (2020). What to do with all the AGREEs? The AGREE portfolio of tools to support the guideline enterprise. J Clin Epidemiol.

[42] Brouwers MC, Spithoff K, Kerkvliet K, Alonso-Coello P, Burgers J, Cluzeau F, Férvers B, Graham I, Grimshaw J, Hanna S, Kastner M, Kho M, Qaseem A, Straus S, Florez ID (2020). Development and validation of a tool to assess the quality of clinical practice guideline recommendations. JAMA Netw Open.

[43] (2019). The Appraisal of Guidelines Research & Evaluation—Recommendation EXcellence (AGREE-REX) [Electronic version]. AGREE-REX Research Team.

[44] Welcome to PADA—a colloborative platform for authors of healthcare decision aids. PADA.

[45] (2015). Gute Praxis Gesundheitsinformationen [Good practice health information]. German Network for Evidence-based Medicine (Deutsches Netzwerk Evidenzbasierte Medizin).

[46] (2005). IPDAS 2005: criteria for judging the quality of patient decision aids. International Patient Decision Aid Standards (IPDAS) Collaboration.

[47] Akl EA, Meerpohl JJ, Elliott J, Kahale LA, Schünemann HJ, Living Systematic Review Network (2017). Living systematic reviews: 4. Living guideline recommendations. J Clin Epidemiol.

[48] (2009). Manual Qualitätsindikatoren [Manual quality indicators]. German Agency for Quality in Medicine (ÄZQ).

[49] (2016). Erstellung von Patientenleitlinien zu S3-Leitlinien/NVL im Rahmen der Leitlinienprogramme [Preparation of patient guidelines on S3 guidelines/NVL within the context of the guideline programs]. German Agency for Quality in Medicine (ÄZQ), Office of the German Guideline Program in Oncology, Association of the Scientific Medical Societies (AWMF).

[50] (2020). FAIR Data Maturity Model: specification and guidelines. RDA FAIR Data Maturity Model Working Group.

[51] Barratt A (2008). Evidence based medicine and shared decision making: the challenge of getting both evidence and preferences into health care. Patient Educ Couns.

[52] Páez G, Forte DN, Gabeiras MDPL (2021). Exploring the relationship between shared decision-making, patient-centered medicine, and evidence-based medicine. Linacre Q.

[53] van Dulmen SA, Lukersmith S, Muxlow J, Santa Mina E, Nijhuis-van der Sanden MWG, van der Wees PJ, G-I-N Allied Health Steering Group (2015). Supporting a person-centred approach in clinical guidelines. A position paper of the allied health community—Guidelines International Network (G-I-N). Health Expect.

[54] Alper BS (2024). Making guidelines computable. Clin Public Health Guidelines.

[55] Vandvik PO, Brandt L, Alonso-Coello P, Treweek S, Akl EA, Kristiansen A, Fog-Heen A, Agoritsas T, Montori VM, Guyatt G (2013). Creating clinical practice guidelines we can trust, use, and share: a new era is imminent. Chest.

[56] Khalil H (2019). Adapting guidelines to local settings. Int J Evid Based Healthc.

[57] Tan NQP, Maki KG, López-Olivo MA, Geng Y, Volk RJ (2023). Cultural influences on shared decision-making among Asian Americans: a systematic review and meta-synthesis of qualitative studies. Patient Educ Couns.

[58] Charles C, Gafni A, Whelan T, O'Brien MA (2006). Cultural influences on the physician-patient encounter: the case of shared treatment decision-making. Patient Educ Couns.

[59] Yates JF, de Oliveira S (2016). Culture and decision making. Organ Behav Hum Decis Process.

