# Supplementary material for: Fostering Shared Decision-Making Between Patients and Health Care Professionals in Clinical Practice Guidelines: Protocol for a Project to Develop and Test a Tool for Guideline Developers
Source: JMIR Res Protoc. 2024 Nov 4;13:e57611. doi: 10.2196/57611 (PMC11574490; doi:10.2196/57611)
Supplement: Multimedia Appendix 1 [file resprot_v13i1e57611_app1.docx]

Search strategy for PubMed:

(method* [tiab] OR approach* [tiab] OR strateg* [tiab] OR procedure* [tiab] OR integrat* [tiab] OR creat* [tiab] OR develop* [tiab] OR framework* [tiab]) AND ("patient decision aid*" [tiab] OR "decision support tool*" [tiab] OR "shared decision making tool*" [tiab] OR "shared decision-making tool*" [tiab] OR "Decision Making, Shared" [mesh] OR "shared decision making*" [tiab] OR "shared decision-making*" [tiab]) AND ("guideline*" [ti]) Filters: from 2000 – 2023

Search strategy used for Embase:

(method*:ab,ti OR approach*:ab,ti OR strateg*:ab,ti OR procedure*:ab,ti OR integrat*:ab,ti OR creat*:ab,ti OR develop*:ab,ti OR framework*:ab,ti) AND ('patient decision aid*':ab,ti OR 'decision support tool*':ab,ti OR 'shared decision making tool*':ab,ti OR 'shared decision-making tool*':ab,ti OR 'shared decision making'/de OR 'shared decision making*':ab,ti OR 'shared decision-making*':ab,ti) AND ('clinical practice guideline'/de OR guideline*:ti) AND ([article]/lim OR [article in press]/lim OR [data papers]/lim OR [review]/lim OR [short survey]/lim OR [preprint]/lim) AND [embase]/lim AND [2000-2024]/py

|  |
| --- |
