# Supplementary material for: Fostering Shared Decision-Making Between Patients and Health Care Professionals in Clinical Practice Guidelines: Protocol for a Project to Develop and Test a Tool for Guideline Developers
Source: JMIR Res Protoc. 2024 Nov 4;13:e57611. doi: 10.2196/57611 (PMC11574490; doi:10.2196/57611)
Supplement: Multimedia Appendix 2 [file resprot_v13i1e57611_app2.docx]

| American Academy of Neurology |
| --- |
| American College of Gastroenterology |
| American College of Physicians |
| American Society of Clinical Oncology |
| American Urological Association, Urology Care Foundation |
| Association of the Scientific Medical Societies |
| Canadian Diabetes Association /Diabetes Canada |
| Canadian Paediatric Society |
| Centers for Disease Control and Prevention |
| European Society for Medical Oncology |
| National Comprehensive Cancer Network |
| German National Healthcare Guidelines |
| National Institute for Health and Care Excellence |
| Programme Oncology |
| Queensland Clinical Guidelines |
| Royal College of Obstetricians and Gynaecologists |
| Scottish Intercollegiate Guidelines Network |
| University of Michigan Hospital and Health Centers |
| UpToDate |
| US Preventive Services Task Force |
|  |
